# Antimicrobial Blue Light for Prevention and Treatment of Highly Invasive *Vibrio vulnificus* Burn Infection in Mice

**DOI:** 10.3389/fmicb.2022.932466

**Published:** 2022-07-12

**Authors:** Carolina dos Anjos, Leon G. Leanse, Xiaojing Liu, Hugo V. Miranda, R. Rox Anderson, Tianhong Dai

**Affiliations:** ^1^Wellman Center for Photomedicine, Massachusetts General Hospital, Harvard Medical School, Boston, MA, United States; ^2^Vaccine and Immunotherapy Center, Massachusetts General Hospital, Harvard Medical School, Boston, MA, United States; ^3^Institute of Photomedicine, Shanghai Skin Disease Hospital, Tongji University School of Medicine, Shanghai, China; ^4^Naval Medical Research Center, Silver Spring, MD, United States

**Keywords:** antimicrobial blue light, bioluminescence imaging, burn infection, cytokines, porphyrins, *Vibrio vulnificus*

## Abstract

*Vibrio vulnificus* is an invasive marine bacterium that causes a variety of serious infectious diseases. With the increasing multidrug-resistant variants, treatment of *V*. *vulnificus* infections is becoming more difficult. In this study, we explored antimicrobial blue light (aBL; 405 nm wavelength) for the treatment of *V. vulnificus* infections. We first assessed the efficacy of aBL against five strains of *V. vulnificus in vitro*. Next, we identified and quantified intracellular porphyrins in *V. vulnificus* to provide mechanistic insights. Additionally, we measured intracellular reactive oxygen species (ROS) production and bacterial membrane permeabilization following aBL exposures. Lastly, we conducted a preclinical study to investigate the efficacy and safety of aBL for the prevention and treatment of burn infections caused by *V. vulnificus* in mice. We found that aBL effectively killed *V. vulnificus in vitro* in both planktonic and biofilm states, with up to a 5.17- and 4.57-log_10_ CFU reduction being achieved, respectively, following an aBL exposure of 216 J/cm^2^. Protoporphyrin IX and coproporphyrins were predominant in all the strains. Additionally, intracellular ROS was significantly increased following aBL exposures (*P* < 0.01), and there was evidence of aBL-induced permeabilization of the bacterial membrane (*P* < 0.0001). In the preclinical studies, we found that female mice treated with aBL 30 min after bacterial inoculation showed a survival rate of 81% following 7 days of observation, while only 28% survival was observed in untreated female mice (*P* < 0.001). At 6 h post-inoculation, an 86% survival was achieved in aBL-treated female mice (*P* = 0.0002). For male mice, 86 and 63% survival rates were achieved when aBL treatment was given 30 min and 6 h after bacterial inoculation, respectively, compared to 32% survival in the untreated mice (*P* = 0.0004 and *P* = 0.04). aBL did not reduce cellular proliferation or induce apoptosis. We found five cytokines were significantly upregulated in the males after aBL treatment, including MCSF (*P* < 0.001), MCP-5 (*P* < 0.01), TNF RII (*P* < 0.01), CXCL1 (*P* < 0.01), and TIMP-1 (*P* < 0.05), and one in the females (TIMP-1; *P* < 0.05), suggesting that aBL may induce certain inflammatory processes. In conclusion, aBL may potentially be applied to prevent and treat *V. vulnificus* infections.

## Introduction

*Vibrio vulnificus* is a Gram-negative and highly pathogenic bacterium found within marine environments (Bross et al., 2007). It can cause a wide array of illnesses ranging from gastroenteritis to skin and soft tissue infections (SSTIs), which often lead to sepsis (Baker-Austin and Oliver, 2018; Yun and Kim, 2018; Centers for Disease Control Prevention, 2019). Frequently, individuals suffering from SSTIs caused by *V. vulnificus* will require limb amputation (Bross et al., 2007; Baker-Austin and Oliver, 2018). In addition, *V. vulnificus* infections are associated with a ≥ 50% mortality rate, with patients suffering from underlying conditions (such as liver disease or immunosuppression) being at most risk (Bross et al., 2007). It has been found that males are more susceptible to developing severe illness from *V. vulnificus* relative to females (Jones and Oliver, 2009). The progression of *V. vulnificus* infections is often rapid, with the development of severe clinical signs (such as sepsis) occurring as early as within 72 h post-exposure (Bross et al., 2007; Baker-Austin and Oliver, 2018). Therefore, it is essential that treatment is initiated promptly to reduce morbidity and mortality. The current standard of care for *V. vulnificus* infections is systemic antibiotic treatment using tetracycline associated with ceftazidime or ciprofloxacin (Tang et al., 2002). However, with the increasing emergence of multidrug-resistant variants, controlling *V. vulnificus* infections is becoming more difficult (Al-Dulaimi et al., 2019; Cutugno et al., 2020).

In recent years, our laboratory has been interested in harnessing the microbicidal effects of antimicrobial blue light (aBL; 405 nm wavelength) as a potential alternative or adjunct treatment to conventional antibiotic approaches (Wang et al., 2017b; Leanse et al., 2022). Our studies have demonstrated that aBL can effectively kill a range of microorganisms without the generation of resistance to aBL becoming a significant concern (Leanse et al., 2018; Marasini et al., 2021). In addition, numerous studies have shown aBL to be compatible with conventional and non-conventional agents, illustrating its potential as an adjunct therapeutic (Leanse et al., 2020a, 2021b; Li and Wu, 2021; Dong et al., 2022a,b). The mechanism of aBL killing of microorganisms is currently understood to be mediated *via* photoexcitation of endogenous chromophores (primarily porphyrins) to induce the production of reactive oxygen species (ROS) that damage the internal structures of the microbial cell (Wang et al., 2017b; Chu et al., 2019). More recently, other molecular targets of aBL have been identified, such as staphyloxanthin in *Staphylococcus aureus* and catalase in numerous microbes, both of which have been shown to sensitize microbes to ROS (Dong et al., 2019, 2022a,b; Hui et al., 2020). aBL elicits its microbicidal effects much more rapidly than traditional antibiotics. Therefore, given the expeditious progression of *V. vulnificus* infections, we considered that aBL may be a more suitable approach to reduce the mortality rate induced by *V. vulnificus*. The primary aim of this study was to investigate the efficacy of aBL for killing *V. vulnificus in vitro* under biologically relevant conditions (i.e., planktonic and biofilm conditions). We then sought to evaluate the potential of aBL to reduce the lethality of *V. vulnificus* infection in a mouse burn model. In addition, we performed a safety assessment on mouse skin following aBL exposure.

## Materials and Methods

### Light Source

For *in vitro* irradiation, aBL was delivered using a single light-emitting diode (LED) with a peak emission of 405 nm and a full width at half-maximum (FWHM) of 25 nm (M405L4; Thorlabs, USA). The LED was positioned at 4 cm above the surface of a sample, and the irradiance level was adjusted to 60 mW/cm^2^, as determined by a PM100D power/energy meter (Thorlabs). For *in vivo* experiments, the LED was positioned at 25 cm above the mouse wound and coupled with a collimator (SM2F32-A; Thorlabs, USA) to focus the light source onto the region of interest, and the irradiance was set to 100 mW/cm^2^.

### Bacterial Strains and Culture Conditions

For *in vitro* efficacy studies, we selected five strains of *V. vulnificus*. Four were obtained from the American Type Culture Collection (ATCC) (ATCC 27562, ATCC BAA-87, ATCC 43382, and ATCC 33817), which were isolated from human blood and leg wound infections, and one was a genetically engineered bioluminescent strain (BCRC 81152) with genotype *pilA luxCDABE*. For the preclinical study in mice, the bioluminescent BCRC 81152 strain was used, allowing real-time monitoring of the extent of infection in mice *via* bioluminescence imaging (Avci et al., 2018). All strains were cultured on marine agar or broth (2216 Difco^TM^, BD, USA) and incubated at 37°C in a stationary or orbital incubator (100 rpm).

### The Killing of Planktonic *V. vulnificus* Using aBL

To assess the aBL efficacy against planktonic *V. vulnificus*, overnight bacterial cultures were centrifuged and washed in phosphate-buffered saline (PBS). The bacterial concentrations were adjusted to ~10^8^ colony-forming units (CFU)/mL by measuring the optical density at λ_abs_ = 600 nm (OD_600−nm_) of the suspensions with a spectrophotometer (Ultrospec 10, Biochrom, USA; OD_600−nm_= 0.1). Subsequently, a 3-ml suspension of each strain was transferred to a 35 mm × 12 m dish and exposed to aBL. During irradiation, the bacterial suspension was stirred using a mini magnetic bar oscillated at 20 rpm, and aliquots (40 μL) of the suspension were withdrawn when 36, 72, 108, 144, 180, and 216 J/cm^2^ aBL exposure had been delivered, reflecting 10, 20, 30, 40, 50, and 60 min of irradiation time, respectively. Aliquots were then subjected to 10-fold serial dilution and plated on agar for CFU enumeration. Experiments were performed in triplicate over 3 days.

### Killing of *V. vulnificus* in Biofilms Using aBL

The efficacy of aBL in killing *V. vulnificus* was also assessed in 48-h-old biofilms *in vitro*. In brief, overnight bacterial cultures were adjusted to a concentration of ~10^6^ CFU/mL. To form biofilms, 100 μl of bacterial suspensions was inoculated in 96-well microtiter plates and incubated at 37°C for 48 h, with a replacement of medium at 24-h intervals (Park et al., 2015). Following 48-h incubation, biofilms were carefully washed twice with PBS and subsequently exposed to increasing aBL exposures of 0, 54, 108, 162, and 216 J/cm^2^, respectively. After aBL exposures, biofilms were washed twice with PBS, scraped in 100 μl of fresh PBS using a 200 μl sterile pipette tip, and then transferred to 1.5 ml microcentrifuge tubes. The procedure was repeated by adding 100 μl of PBS, followed by 5 min of sonication (M2800; Branson Ultrasonics, USA) to disperse bacterial biofilm (Ferrer-Espada et al., 2019). CFU was quantified through serial dilution and plating on marine agar. Experiments were performed in triplicate over 3 different days.

### Transmission Electron Microscopy of *V. vulnificus* Following aBL Exposure to Assess Ultrastructural and/or Morphological Changes

Transmission electron microscopy (TEM) was performed to determine ultrastructural and/or morphological alterations in *V. vulnificus* induced by aBL exposure. In this study, ATCC 27562 was selected as the representative strain, and two aBL exposures of 108 and 216 J/cm^2^ were studied. An untreated sample was used as a negative control. Following the treatments, samples were fixed in 2.5% glutaraldehyde and 2% paraformaldehyde and stored overnight at 4°C. The samples were then centrifuged and washed twice in 0.1 M sodium cacodylate buffer (pH 7.2), and the pellets were post-fixed in 2% ostium tetroxide buffer for 8 h. Samples were then serially dehydrated in ethanol and embedded in resin (Tousimis, USA). Ultrathin sections (<100 nm) were cut by using diamond blades of an ultramicrotome, collected on uncoated 200-mesh copper grids, and stained with uranyl acetate and lead citrate. The images were captured on a Philips CM-10 TEM (Philips Electronics, USA).

### Identification and Quantification of Endogenous Porphyrins in *V. vulnificus* by Using Ultra-Performance Liquid Chromatography

The ultra-performance liquid chromatography (UPLC) analyses were performed to identify and quantify the endogenous porphyrins present in the *V. vulnificus* strains. In brief, bacteria in overnight cultures (20 ml cultures × 3 in 50 ml tubes) were harvested by centrifugation (10 min at 4,000 rpm), resuspended into 1 ml of extraction solution (ethanol, dimethyl sulfoxide, acetic acid, 80: 20: 1, vol/vol/vol), and stored at −80°C for 24 h (Wang et al., 2019b). After incubation, bacterial cells were disrupted in an ultrasonic bath (M2800; Branson Ultrasonics, USA) for 30 min. Samples were then centrifuged, and the supernatant was collected for UPLC analysis. Chromatographic marker kits that enable the detection of uroporphyrin, heptaporphyrin, hexaporphyrin, pentaporphyrin, coproporphyrin, and protoporphyrin IX were used. The results were compared with chromatographs produced by standard porphyrins. The analysis was carried out using a Waters® Acquity UPLCTM system.

### Detection of General Reactive Oxygen Species in *V. vulnificus* Induced by aBL Exposure

To measure the intracellular reactive oxygen species (ROS) promoted by aBL exposure, we used the probe 2′, 7′-dichlorofluorescein diacetate (DCFH-DA; D6883, Sigma-Aldrich, USA). The DCFH-DA is a permeable probe that does not emit fluorescence; however, it becomes increasingly fluorescent once oxidized to 2',7' dichlorofluorescein (DCF). In this study, the DCFH-DA was added to the bacterial suspension (10^9^ CFU/mL) at a final concentration of 20 μM and incubated at room temperature (~25°C) for 30 min to permit permeation of the probe (Wu et al., 2018). Following the incubation, 3 ml of samples was transferred to a 35 × 12 mm dish and exposed to increasing aBL radiant exposures of 36, 72, 108, 144, 180, and 216 J/cm^2^. The samples were immediately transferred to 96-well plates after aBL exposures for fluorescence measurement at λ_ex_ = 504 nm and λ_em_ 529 nm, using a Spectramax plate reader (M4, Molecular Devices, USA).

### Membrane Permeability Following aBL Exposure

To verify if aBL can facilitate membrane damage in *V. vulnificus*, we performed the propidium iodide (PI) permeabilization assay. PI is a fluorochrome that binds to DNA strands but cannot pass through a healthy cytoplasmic membrane. We selected *V. vulnificus* ATCC 27562 as the representative strain in this study. Bacterial suspensions were prepared as described above and transferred to a 35 mm × 12 mm dish for aBL exposures (i.e., 36, 72, 108, 144, 180, and 216 J/cm^2^). The negative control was an untreated sample, and the positive control of membrane damage was a sample pre-incubated with 70% ethanol for 15 min. After aBL exposures, the samples were incubated with PI (50 μg/mL; BD Pharmingen, USA) in the dark for 15 min in 96-well plates. The PI uptake was measured using a microplate spectrophotometer (SpectraMax M4; Molecular Devices, USA) at λ_ex_ = 520 nm and λ_em_ 620 nm.

### *V. vulnificus* Burn Infection in Mice

BALB/c mice, 7- to 8-week-old and weighing 17–22 g, were obtained from Charles River Laboratories (Wilmington, MA). To consider sex as a biological variable, we included both male and female mice in the study. Five animals were housed per cage, with water and food *ad libitum*, and maintained on a 12-h dark-light cycle at 21°C and 30–70% relative humidity. The procedures on animals were in accordance with the National Institute of Health (NIH) and approved by the Institutional Animal Care and Use Committee (IACUC) of the Massachusetts General Hospital.

Before creating burn injuries, mice were anesthetized by intraperitoneal (i.p.) injection of ketamine–xylazine (100–20 mg/kg). Ten (10) min after anesthesia, the animals were shaved in the dorsal area using a 50-blade hair clipper. Burn injuries were created by placing a preheated brass block (~1 cm^2^ in cross-section; ~100°C) on the shaved sacral-dorsal surface of mice for 3 s, resulting in third-degree burns. Five min after burning, each burned area was inoculated with a 100-μl suspension containing ~10^7^ CFU of bioluminescent *V. vulnificus* (BCRC 81152). Buprenorphine (0.1 mg/kg; i.p) was applied twice daily for 3 days as a pre-emptive and postoperative analgesic for pain relief. Mouse eyes were covered using eye ointment to prevent dryness during anesthesia (Wang et al., 2017a).

### aBL Efficacy for Prevention and Treatment of *V. vulnificus* Burn Infection in Mice

To assess the efficacy of aBL in the prevention (or post-exposure prophylaxis) of *V. vulnificus* burn infections in mice, aBL was initiated 30 min after bacterial inoculation. To study the efficacy of aBL in the treatment of infection, aBL was started 6 h after bacterial inoculation, as our preliminary studies showed that a period of 6 h was sufficient to establish *V. vulnificus* infection in mice (Supplementary Figure 1). The severity of infection in mice was quantified by measuring the intensity of the bioluminescence signal [relative luminescence units (RLU)] from the photon emission by *V. vulnificus* using an IVIS Lumina II In Vivo Imaging System (PerkinElmer, Waltham, MA). In a subset of mice (*n* = 2), the correlation between RLU and CFU was determined following exposure to varying aBL intensities of 0, 15, 30, 60, 120, and 360 J/cm^2^. For both conditions (i.e., prevention or treatment of infection), aBL was delivered with radiant exposures of 0, 30, 60, 90, 120, 150, 180, and 360 J/cm^2^, reflecting 0, 5, 10, 15, 20, 25, 30, and 60 min of irradiation time (irradiance = 100 mW/cm^2^), respectively. Bioluminescence imaging was performed after each exposure to light. After aBL treatment, mice were monitored for survival for up to 7 days.

### Measurement of Proinflammatory Cytokines in Mouse Skin After aBL Exposure

Mice, including both males and females, were exposed to 360 J/cm^2^ aBL at 100 mW/cm^2^ irradiance on naïve skin (i.e., healthy skin, non-burned, and non-infected), reflecting the maximal therapeutic dose used in the treatment of *V. vulnificus* infections. At 24 h following aBL exposure, the animals were euthanized, and their skin was sampled for a proinflammatory panel, which included the measurement of 40 cytokines: BLC, CD30L, Eotaxin, Eotaxin-2, Fas L, G-CSF, GM-CSF, ICAM-1, IFNg, IL-1a, IL-1b, IL-2, IL-3, IL-4, IL-5, IL-6, IL-7, IL-10, IL12p70, IL-13, IL-15, IL-17, IL-21, KC, Leptin, LIX, MCP-1, MCP-5, MCSF, MIG, MIP-1a, MIP-1g, PF4, RANTES, TARC, TCA-3, TIMP-1, TNFa, TNF RI, and TNF RII. In brief, mouse skin tissue was homogenized in a tube (Lysing Matrix D, MP Biomedicals) with 1 ml of 1x lysis buffer supplemented with Phosphatase/Protease Inhibitor Set (AA-PIPHI, RayBiotech, Norcross, GA) for use with ELISA kits (RayBiotech, Norcross, GA). The proinflammatory cytokine profile was measured using the Quantibody Mouse Inflammation Array Q1- RayBiotech (RayBiotech, USA).

### Cell Proliferation and Apoptosis Activity in Mouse Skin After aBL Exposure

In this study, naïve skin in mice was first exposed to aBL at 360 J/cm^2^, and then mice were euthanized at 24 h or 72 h following aBL exposure. Treated mouse skin was harvested and fixed in 10% phosphate-buffered formalin (Fisher Scientific, USA) for 72 h before immunohistochemical processing. Mice that were not treated were also included. To determine the effect of aBL on cell proliferation, a Ki-67 antibody (ab 16667; Abcam, USA) conjugated to a probe labeled with fluorescent Cy3 (λ_ex_ = 578 nm and λ_em_ = 603 nm) was used. Cells that actively expressed Ki-67 were considered to be actively proliferating cells (Guinebretière and Sabourin, 1997). For apoptosis determination, a caspase-3 antibody (966; Cell Signaling, USA) conjugated to a fluorescent Cy5 labeled probe was used (λ_ex_ = 658 nm and λ_em_ = 675 nm). Apoptosis was observed in the cells expressing caspase-3 protein (Mazumder et al., 2008). Immunofluorescence sections were imaged using the Nanozoomer S60 digital slide scanner (C13210-01; Hamamatsu, USA). Three regions of interest (ROI; the pixelated area was set to 7.3 × 10^5^) were selected randomly from the images of three different mice, respectively, to determine the percentage fluorescence of Ki-67 (at 578 nm) or caspase-3 (at 675 nm) relative to the entire analyzed area (Jensen, 2013) by using Image J. Additionally, hematoxylin-eosin (H&E) staining was performed to qualitatively assess any cellular or structural skin changes.

### Statistical Analyses

All *in vitro* experiments were performed in triplicate. In the animal study, the sample size of animals (*n* = 22) was determined by using a power analysis. Quantitative data were presented as mean ± standard error of the mean (SEM). Statistical analysis was performed using a one- or two-way analysis of variance (ANOVA) with Dunnett's and Tukey's *post-hoc* comparisons, respectively. The log-rank test was performed for animal survival analysis, and the unpaired *t*-test was done to compare the cytokine concentrations between groups. All statistical analyses were performed by using GraphPad Prism 9.0 software (GraphPad, USA). Values of *P* < 0.05 were considered statistically significant.

## Results

### aBL Effectively Killed *V. vulnificus* in Both Planktonic and Biofilm States

**Figure 1:**
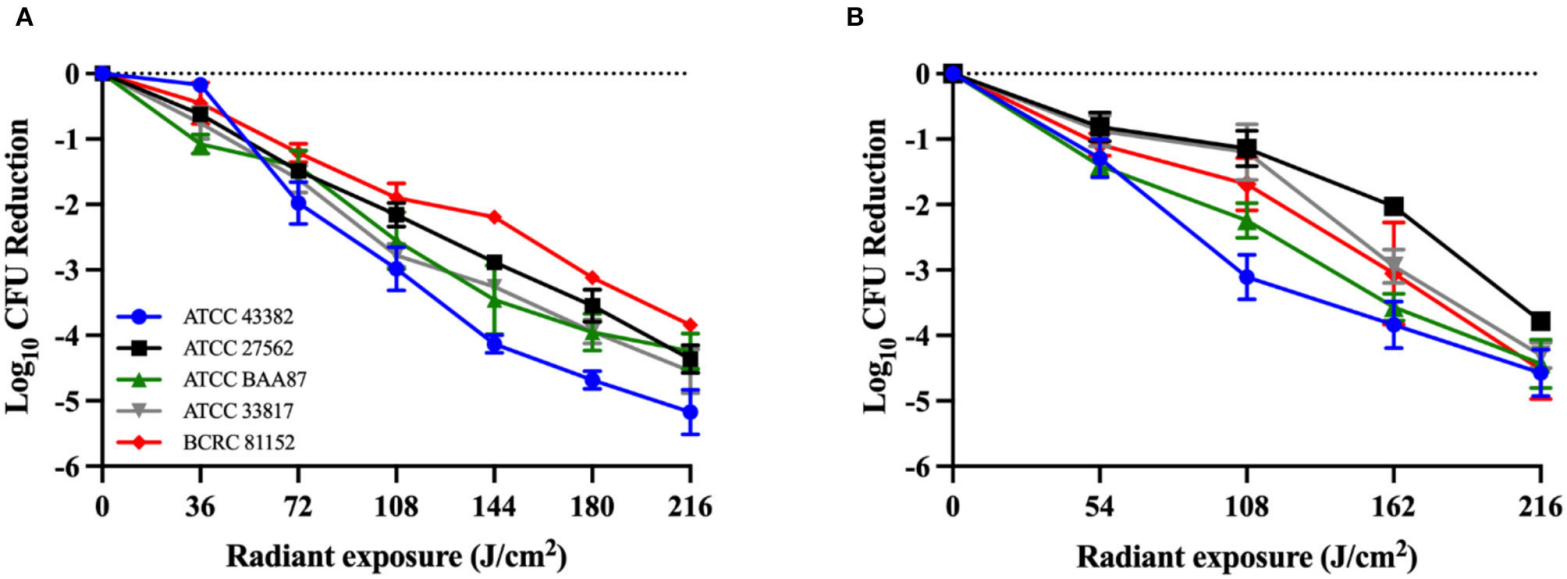
Killing kinetics of *Vibrio vulnificus* by antimicrobial blue light (λ = 405 ± 25 nm) in **(A)** planktonic bacteria and **(B)** 48-h-old biofilms. All experiments were performed in triplicate. Data are presented as the mean and standard error of the mean (SEM) values.

Antimicrobial blue light killed all *V. vulnificus* strains studied in a dose-dependent manner in both planktonic and biofilm cultures (Figures 1A,B). Although no significant differences were observed between the killing kinetic curves of different strains, differences in the killing efficiencies were observed at certain aBL exposure levels. For planktonic cultures, ATCC 43382 was found to be the most sensitive to aBL, reaching an average of 5.17-log_10_ CFU reduction after 216 J/cm^2^ light exposure, relative to untreated control. BCRC 81152 was identified to be the most tolerant strain, with 3.84-log_10_ CFU reduction being observed. For *V. vulnificus* biofilms, ATCC 43382 also exhibited the highest susceptibility to killing by aBL. After an aBL exposure of 216 J/cm^2^, an average of 4.57-log_10_ CFU reduction was achieved in ATCC 43382, relative to an untreated control sample. The most aBL-tolerant strain in biofilm was ATCC 27562, with 3.58-log_10_ CFU reduction being observed following an equivalent aBL exposure. There was some discrepancy between the most sensitive strain against planktonic vs. biofilms, which may be due to variable biofilm formation. Further work assessing the biofilm formation potential of these strains may be warranted to elucidate this observation.

### aBL Induced Ultrastructural and Morphological Changes in *V. vulnificus*

**Figure 2:**
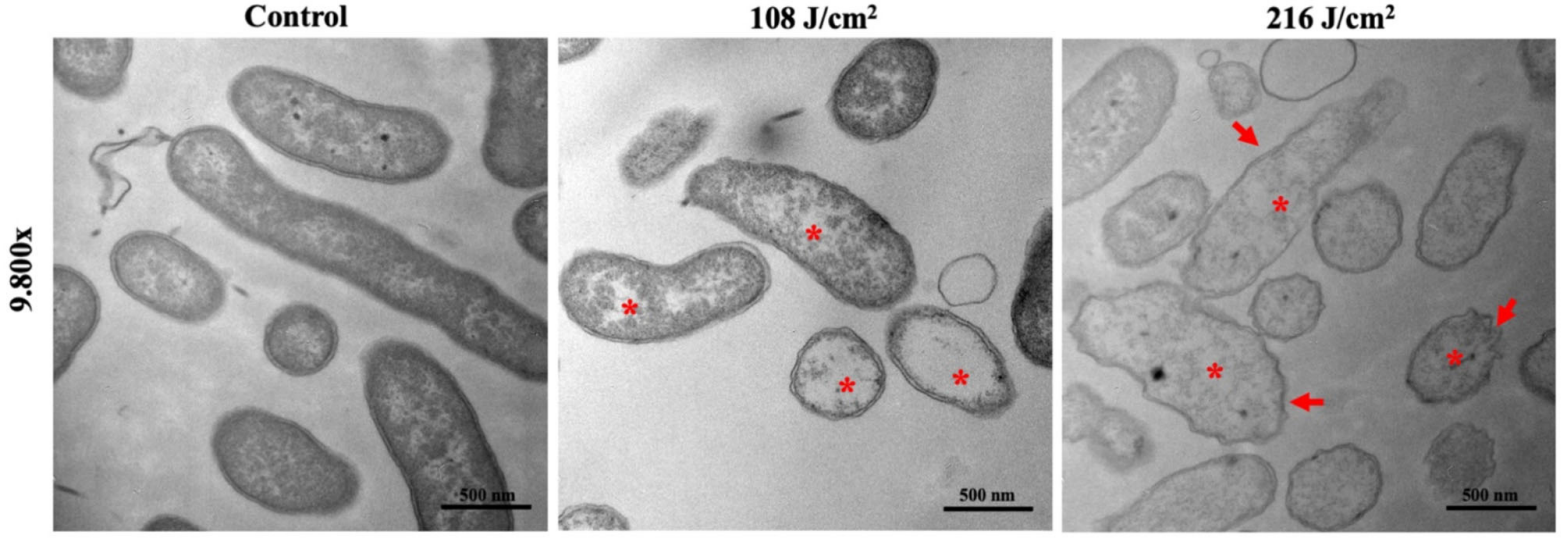
Representative transmission electron microscopy images illustrate ultrastructural and morphological damages in *Vibrio vulnificus* following antimicrobial blue light exposures (108 and 216 J/cm^2^). **Asterisk:** cytoplasmatic vacuoles and bubbles; **arrows:** membrane irregularities. Bars: 500 nm.

The TEM images showed ultrastructural damages in *V. vulnificus* after 108 or 216 J/cm^2^ aBL exposure (Figure 2). Membrane irregularities (**red arrow**) and reduced cytoplasmic density (**red asterisk**) were observed, with the presence of intracellular aggregation and vacuole formation.

### Porphyrins Were Present in *V. vulnificus*

**Figure 3:**
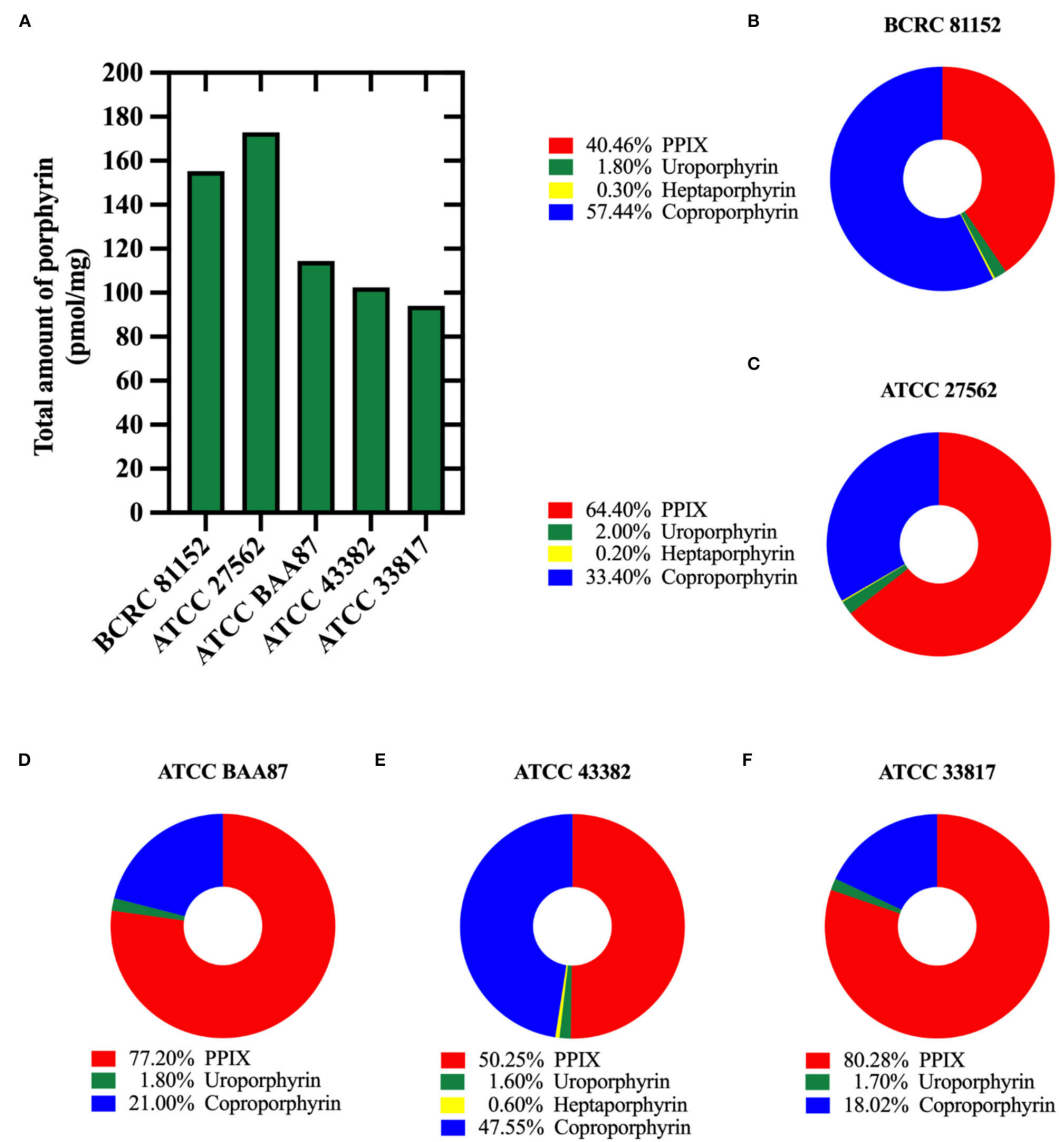
Quantification of endogenous porphyrins in *Vibrio vulnificus*. **(A)** The total amount of porphyrins (pmol/mg of bacterial protein) in different *V. vulnificus* strains. **(B–F)** Percentages of different porphyrin species identified in: **(B)** BCRC 81152, **(C)** ATCC 27562, **(D)** ATCC BAA87, **(E)** ATCC 43382, and **(F)** ATCC 33817.

We identified and quantified the endogenous porphyrins present in all five (5) *V. vulnificus* strains studied. BCRC 81152 strain showed the highest total amount of endogenous porphyrins (155.24 pmol/mg), while ATCC 33817 strain contained the lowest total porphyrin amount (94.01 pmol/mg) (Figure 3A). As shown in Figures 3B–F, protoporphyrin IX and coproporphyrins were predominant in all the strains, with protoporphyrin IX being the most prevalent in four out of the five strains. Coproporphyrin was the most prevalent in BCRC 81152. All the strains were also marked with modest concentrations of uroporphyrins and/or heptaporphyrins, but no amounts of pentaporphyrins and hexaporphyrins were detected.

### aBL Induced ROS Production and Membrane Damage in *V. vulnificus*

**Figure 4:**
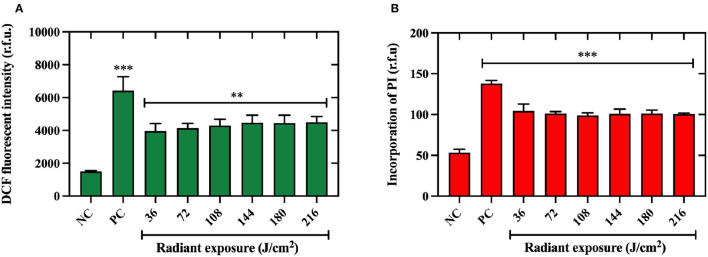
Quantification of reactive oxygen species production and membrane permeabilization in *Vibrio vulnificus* following aBL. **(A)** Intracellular reactive oxygen species (ROS) detected in *V. vulnificus* ATCC 27562 after antimicrobial blue light (aBL) exposure to 2′-7′ dichlorofluorescein (DCF) fluorescent intensity. **(B)** Membrane damage assessed by incorporation of propidium iodide by *V. vulnificus* after aBL exposure. Values are presented as mean ± SEM. *P < 0.05, **P < 0.01, ***P < 0.001 compared to untreated control. NC, negative (untreated) control, PC, positive control.

In this study, we used ATCC 27562 as the representative strain due to insignificant differences in killing efficacy by aBL that were observed amongst the strains studied. We found that even at the lowest light exposure of 36 J/cm^2^, a significant increase in ROS production in bacteria was observed (*P* < 0.01), holding a plateau in DCF detection under higher light exposures up to 216 J/cm^2^ (Figure 4A). Additionally, we found that the low aBL exposure of 36 J/cm^2^ was sufficient to significantly permeabilize the membrane relative to the untreated control (*P* < 0.001; Figure 4B). No further increases in membrane permeabilization were observed under higher aBL exposures.

### aBL Significantly Reduced the Bacterial Burden of *V. vulnificus* Infection in Mice and Improved the Survival of Mice

**Figure 5:**
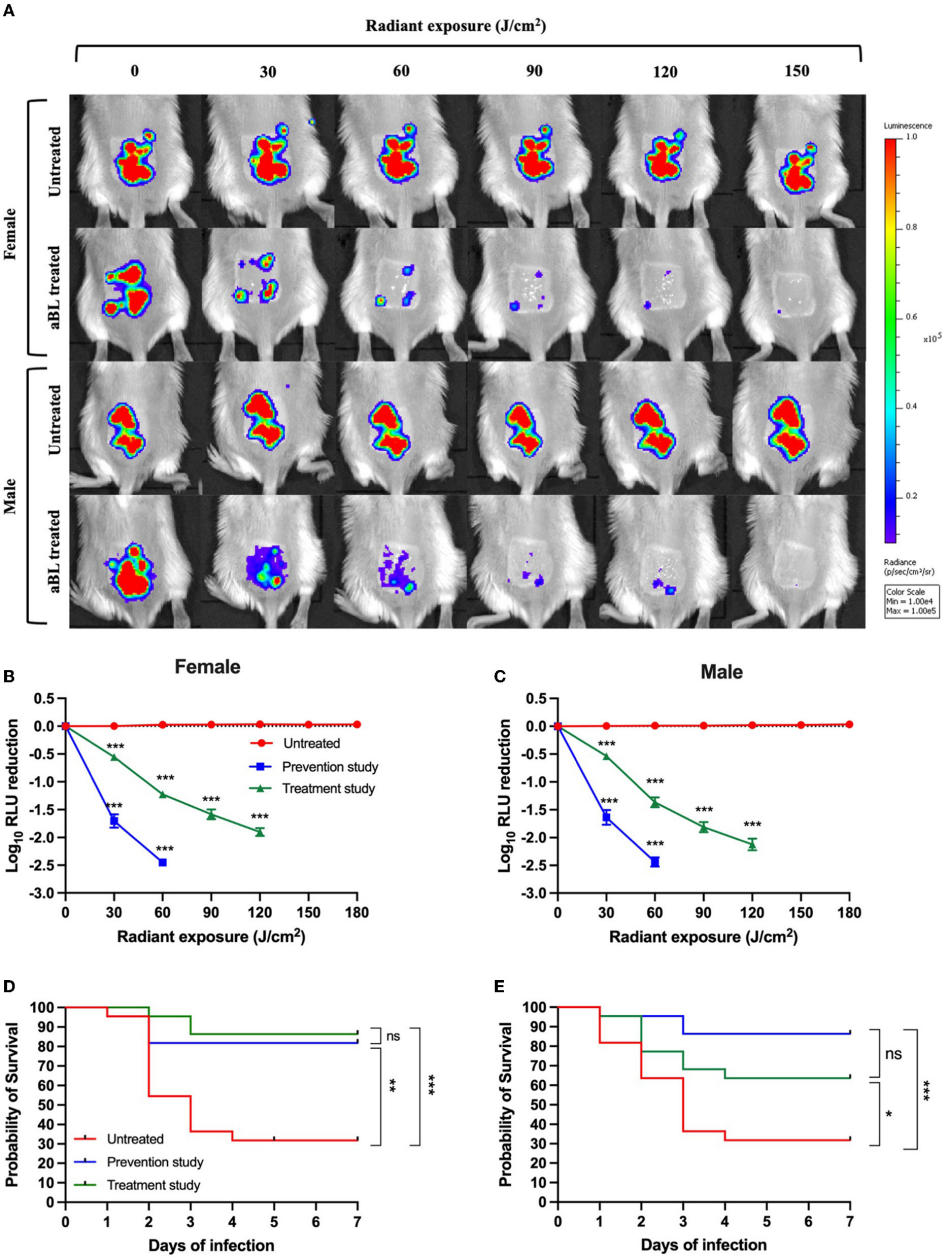
**(A)** Representative bioluminescence images showing the real-time viability of *V. vulnificus* in mice. **(B,C)** Killing kinetics of *V. vulnificus* by antimicrobial blue light (aBL) in female and male mice when aBL was initiated at 30 min and 6 h after bacterial inoculation, respectively, compared to untreated animals (*n* = 22). **(D,E)** Kaplan–Meier survival curves of mice exposed to aBL 30 min and 6 h after bacterial inoculation and untreated mice [**(D)** females and **(E)** males]; *n* = 22. *P < 0.05, **P < 0.01, ***P < 0.001.

Initially, we determined the correlation between *V. vulnificus* RLU and CFU *in vivo*, following different aBL radiant exposures. We found a significant linear correlation between the RLU and CFU (*R*^2^ = 0.94; *P* < 0.001), which indicates that a change in bioluminescence is a true indication of a change in the viability of *V. vulnificus in vivo* (Supplementary Figure 2). Next, using bioluminescence imaging, we assessed the efficacy of aBL in the prevention and treatment of *V. vulnificus* burn infection in mice by initiating aBL exposure at 30 min and 6 h after bacterial inoculation, respectively (Figures 5A–C). In the prevention study, we found a light exposure of 60 J/cm^2^ was sufficient to reduce RLU (or bacterial load) in mouse burns by 2.45-log_10_ units, with similar results being observed with respect to males and females. Unsurprisingly, in the treatment study, the radiant exposure of aBL required to reduce equivalent bacterial load was higher, with 120 J/cm^2^ exposure resulting in a 2-log_10_ RLU reduction in both males and females.

Additionally, we looked at the survival rate of mice following a maximum aBL exposure of 360 J/cm^2^. This light exposure was selected, given that our initial study demonstrated that even at low CFU levels (~10^2^ CFU/wound), which was below the detection limit of bioluminescence imaging, *V. vulnificus* was shown to be lethal in mice. Thus, a higher aBL exposure may be necessary to further reduce the bacterial burden beyond what is detectable *via* bioluminescence imaging (which can only detect ≥ 10^4^ RLU, corresponding to ~10^4^ CFU/wound).

For female mice, the absence of treatment resulted in a survival rate of 28% following 7 days post-inoculation with *V. vulnificus* (Figure 5D). When mice were treated for 30 min after bacterial inoculation (prevention study), the survival rate was increased to 81% (*P* < 0.001). Impressively, in the treatment study (when aBL was initiated 6 h post-inoculation), the survival rate was 86% following 7 days of monitoring (*P* < 0.001). There was no statistically significant difference in the survival rate between the prevention and treatment groups following 7 days of observation (*P* = 0.56).

For male mice, the findings were similar to those of females, with untreated mice presenting a survival rate of 32% following 7 days of post-inoculation (Figure 5E). The survival was increased to 86% in the prevention study (*P* = 0.0004) and 63% in the treatment study (*P* = 0.04) (Figure 5E). No statistically significant difference in the survival rate was found when comparing prevention and treatment groups following 7 days of observation (*P* = 0.08).

### Cytokine Analyses Revealed Inflammatory Responses Trigged by aBL

**Figure 6:**
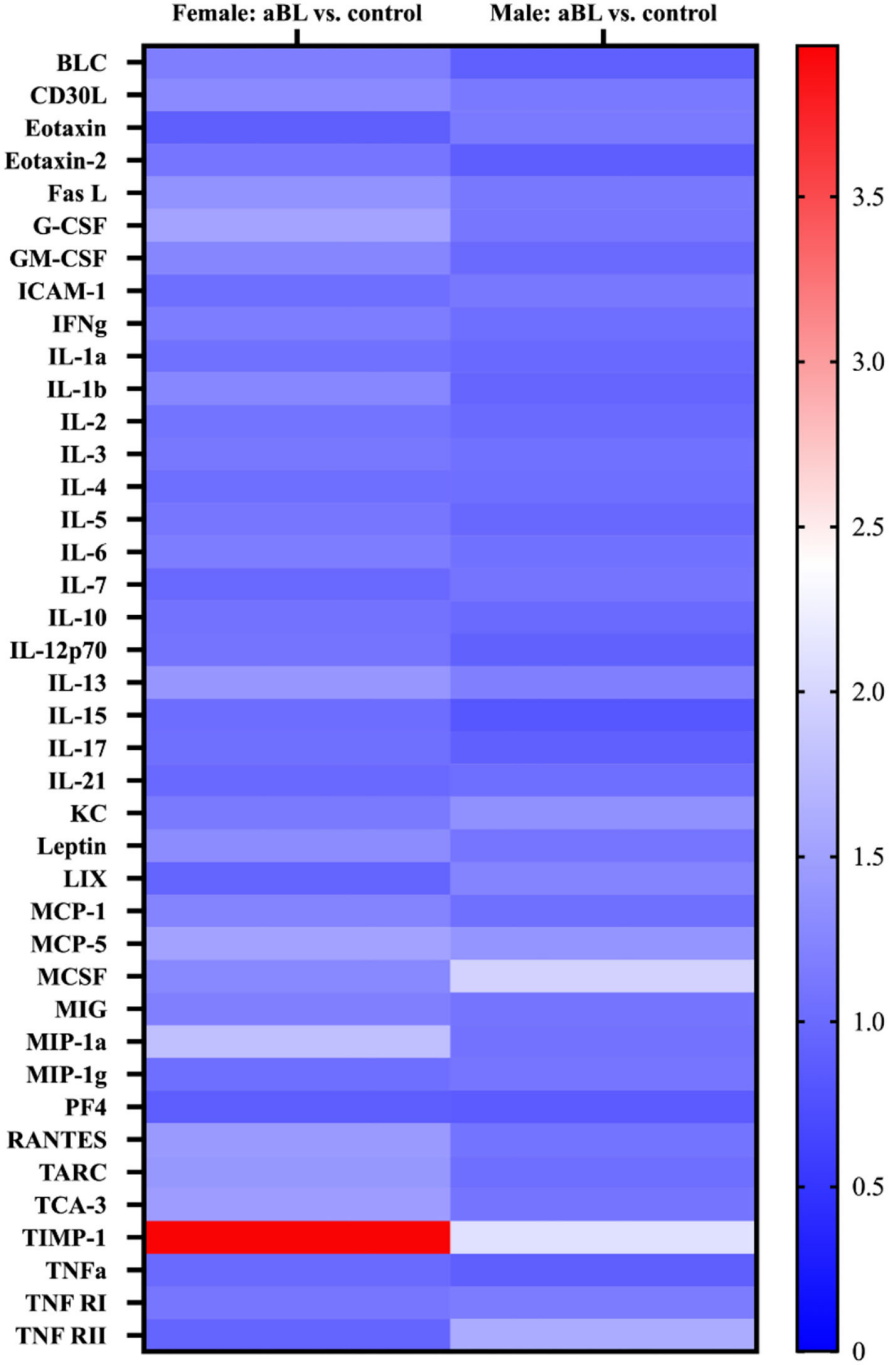
Heatmap display of inflammatory cytokine profile in mouse skin following antimicrobial blue light. Cytokine expression levels are represented by color: blue, lowest expression; white, moderate expression; red, highest expression.

Here, we quantified the concentrations of proinflammatory cytokines in mouse skin 24 h after exposure to aBL. We did not observe any statistically significant changes in the concentrations of most cytokines assessed following aBL irradiation, relative to the untreated control (Figure 6; Supplementary Figure 3). For female mice, aBL induced a significant increase of concentration only in TIMP-1 (*P* < 0.05). For males, the increase in the concentration was observed in MCSF (*P* < 0.001), MCP-5 (*P* < 0.01), CXCL1 [KC] (*P* < 0.01), and TIMP-1 (*P* < 0.05). When determining the fold-changes of different cytokines, we did not observe any striking changes (fold-change above 2) in most of the cytokines when aBL-treated mice were compared with the untreated control. However, the TIMP-1 cytokine was upregulated with a fold-change of 3.95 and 2.08 in the female and male mice, respectively.

### aBL Did Not Significantly Influence Cellular Proliferation or Induce Apoptosis in naïve Mouse Skin

**Figure 7:**
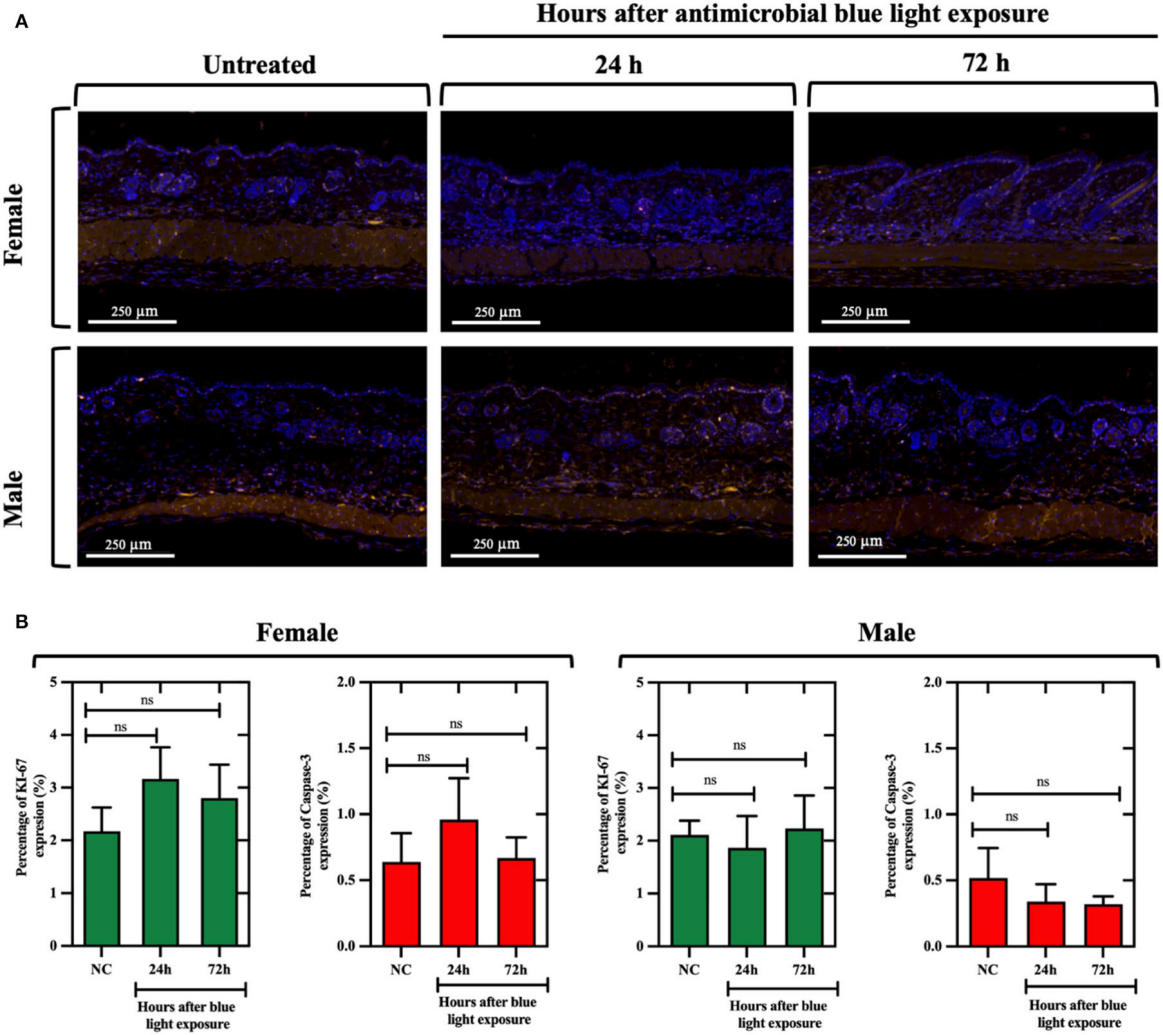
**(A)** Immunofluorescence images of naïve skin in mice exposed to antimicrobial blue light (360 J/cm^2^) using Ki-67 (at 578 nm; yellow) and caspase-3 (at 675 nm; red) antibodies for cell proliferation and apoptosis quantification, respectively. Skin samples were collected at 24 and 72 h after light exposure and counterstained with DAPI (blue). White bar: 250 μm. **(B)** Percentage of Ki-67 and caspase-3 expression relative to the analyzed areas in female and male mouse samples. ns, not significant.

We found that, for both males and females, aBL did not significantly alter the proportion of Ki-67-positive cells (determined at 24 or 72 h post-exposure) relative to the untreated control (Figures 7A,B), as evidenced by similar percentages of fluorescence-expressing (or the Ki-67-positive) cells being observed amongst the groups. For example, in females at 24 h post-exposure, the percentage of Ki-67-positive cells in the untreated mice was 2.17%, and the aBL-treated group exhibited 3.16% of Ki-67-positive cells. While the percentage of Ki-67-positive cells appeared to be slightly higher in the treated group, thus suggesting increased proliferation, this was not found to be statistically significant (*P* =0.41). These observations were comparable at 72 h post-exposure, and findings were similar in the male groups.

With respect to caspase-3-positive cells, fluorescence imaging suggested that aBL did not increase apoptosis in mouse skin, as evidenced by a similar percentage of caspase-3-positive cells found within the untreated and treated groups. For example, following 24 h post-exposure, the percentage of caspase-3-positive cells in the females was 0.63% in the untreated control vs. 0.95% in the aBL-treated samples (*P* = 0.56). Also, the findings at 72 h were similar. In male mice, there was no evidence of increased apoptosis at either 24 h (*P* = 0.64) or 72 h (*P* = 0.59) post-aBL exposure. Similarly, H&E analyses did not reveal any aBL-induced changes in the overall structure and morphology (at 24 or 72 h post-aBL exposure) of the skin relative to the untreated control (Supplementary Figure 4).

## Discussion

Over the past decade, our laboratory has been interested in exploring the potential of aBL as a stand-alone or adjunct therapy against localized infections (Dai et al., 2013; Leanse et al., 2018, 2021a). In the present study, we explored, for the first time, the applicability of aBL against the highly invasive and pathogenic bacterium, *V. vulnificus*. Specifically, we determined the effectiveness of aBL to prevent and treat *V. vulnificus* wound infection in mice with the specific intention to reduce the mortality rates of the animals. In addition, we evaluated the safety of aBL through an assessment of inflammatory cytokine expression, the proliferation of skin cells, and apoptosis.

Initially, we found that *V. vulnificus* in suspensions was highly susceptible to killing by aBL, with up to 5-log_10_ CFU reduction following a radiant exposure of 216 J/cm^2^. This was not surprising, given that a previous study found *Vibrio cholerae* to be highly responsive to blue light (Tardu et al., 2017). What is of more interest is that *V. vulnificus* biofilms appeared to be highly susceptible to aBL, with an analogous sensitivity to that of planktonic *V. vulnificu*s. Previous studies have shown that aBL is less effective in killing microorganisms within biofilms relative to their planktonic counterparts, which is anticipated given that microorganisms are well-known to be more tolerant to conventional antimicrobials when present in a biofilm (Leanse et al., 2020b). Therefore, the relatively high susceptibility of *V. vulnificus* within biofilms (and in the planktonic state) to killing by aBL may be a result of blue light-specific photoreceptors, for example, blue light using flavin (BLUF), that promote ROS production (Plavskii et al., 2018). In a study by Tardu et al. (2017), the authors suggested that aBL-induced ROS production in *V. cholerae* may promote transcriptional upregulation of the *phr* gene, which is involved in genome repair and is photoprotective against ultraviolet irradiation. Therefore, there exists a possibility that the evolutionary requirement for *Vibrio* spp. to react to aBL (and produce ROS) may explain why *V. vulnificus* biofilms generally react more efficiently to aBL exposure, relative to other (non-marine) bacteria. However, further experimental evidence is required to corroborate our hypothesis.

Transmission electron microscopy imaging revealed specific changes in the morphology of *V. vulnificus*, with suggested damage to the cytoplasmic region and cell wall. This finding is in concordance with numerous other studies of aBL, which observed similar changes within different microbial cells. Interestingly, we found that damage elicited following light exposure to 108 J/cm^2^ was similar in nature to the damage observed when the sample was exposed to 216 J/cm^2^, with the exception that there was no evidence of membrane or cell wall irregularities. This suggested that higher radiant energy is necessary to facilitate further damage to each individual cell. Further quantitative studies would be necessary to better understand the relationship between aBL exposure and cellular damage at the level of a single cell.

For several years, intracellular chromophores, primarily porphyrins, have been implicated as inducers of phototoxicity by aBL. Therefore, we quantified the relative concentrations of the intermediate porphyrins and terminal porphyrin PpIX within *V. vulnificus*. As with previous studies (Wang et al., 2019a; Leanse et al., 2021a), we found a detectable amount of porphyrins within all the strains studied, with coproporphyrin and PpIX being the most dominant porphyrin species. These findings are not surprising, given that a previous study by Bumah et al. (2020) found that when a non-porphyrin containing bacterial species *Streptococcus agalactiae* (which is insensitive to aBL-mediated killing) was supplemented with PpIX and coproporphyrin, they were highly susceptible to aBL activity. We did not, however, observe any correlation between the concentrations of porphyrins and the killing potential by aBL, which might suggest that other chromophores (flavins, etc.) may be responsible for promoting antimicrobial effects. Further studies evaluating the effect of aBL on isogenic mutants of *V. vulnificus* that are devoid of porphyrin production would be required to validate their contribution to aBL efficacy.

Previous studies have shown that aBL induces ROS production and membrane damage within bacteria (Wu et al., 2018; Hyun et al., 2021). Therefore, we sought to determine whether *V. vulnificus* was susceptible to these phenomena. Indeed, a statistically significant increase in ROS production was induced following the aBL exposure to 36 J/cm^2^, the lowest aBL exposure we tested. This finding was anticipated, given that aBL-induced ROS production in *V. cholerae* (as well as other bacterial species) has been documented (Tardu et al., 2017). Impressively, uptake of propidium iodide was promoted in aBL-exposed *V. vulnificus*, even at radiant exposures as low as 36 J/cm^2^, suggesting that aBL facilitates permeabilization of the cell membrane and/or cell wall. This is an observation that has been documented in other species and strains (Wu et al., 2018; Hyun et al., 2021). An important consideration, however, is that PI can pass through dead cells (given that selective permeability is lost when cells are killed), which could signify that permeability may also be a result of cell death. Further studies looking at the methods that are able to differentiate the membrane permeability due to aBL exposure from that due to cell death are warranted.

We next evaluated the clinical applicability of aBL for the treatment of burn infections caused by *V. vulnificus* in a mouse model. It is well-known that infections caused by *V. vulnificus* are highly invasive with a high incidence of mortality in patients and have also been shown to be lethal in mice (Tang et al., 2002). In our study, we determined whether aBL could improve the survival rate of mice infected with *V. vulnificus*. We initially established that a 6-h incubation time was sufficient to produce an active *V. vulnificus* burn infection (Supplementary Figure 1), with a large proportion of mice not surviving following 48 h without treatment. It is well-known that males are more at risk of death following *V. vulnificus* infection, which led us to evaluate the efficacy of aBL for the prevention and treatment of *V. vulnificus* infections in both male and female mice. We found that, for females, the survivability was significantly increased following a single exposure of aBL for both 30-min- and 6-h-old infections, due to the rapid action of aBL, suggesting a relatively wide window for aBL treatment after exposure to *V. vulnificus*. This is particularly important in a clinical situation given the rapid progression of severe *V. vulnificus* infection. Therefore, the prompt administration of aBL may provide patients with an extended opportunity to receive antibiotic treatment in a timely fashion. This is further benefited by the potential synergistic interaction between aBL and other antimicrobial substances that may promote more effective treatment of *V. vulnificus* infection. Interestingly, Wong et al. (2005) observed a survival rate of 50% in mice treated with toluidine blue-mediated photodynamic therapy, even though systemic infection had become established. The authors suggested that PDT could effectively downregulate bacterial virulence factors and decrease virulence in the treated animals. This observation could explain our favorable results even when the treatment was started 6 h after bacterial inoculation.

The survival rate of male mice also increased significantly when aBL was delivered at 30 min post-inoculation, in a similar manner as compared to the females. However, when male mice were exposed to aBL 6 h after inoculation, the survival rate was lower compared to that of the females. One potential explanation for the reduced ability of aBL to prevent death in male mice with 6-h-old infections could be due to the relatively greater skin thickness of male mice as compared to females. From our measurements, we found that the female mouse skin has a total thickness of ~370 μm, which was found to be significantly shallower than its male counterpart, which measured 450 μm (*P* < 0.0001; Supplementary Figure 5). It is well-documented that the penetration potential of visible light at 400 nm is ~400 μm (Barolet, 2008), which suggests that the total depth of female mouse skin would be efficiently irradiated. However, for male mice, when bacteria migrated deeper into the skin (>400 μm), they would be beyond the reach of aBL. Further study would be necessary to confirm this. This likely would have consequences when considering a human patient. The limited penetration of aBL in the human tissues, coupled with the invasiveness of *V. vulnificus*, may render the treatment of more established *V. vulnificus* infections difficult. Therefore, the use of optical clearing agents and/or microneedle arrays for the interstitial administration of aBL may be required to ensure improved exposure of the invading bacteria to aBL (Kim et al., 2016; Wang et al., 2021; Tuchin et al., 2022).

To explore the possible molecular biological influence of applying aBL as a treatment for *V. vulnificus* infections, we first evaluated whether there was any significant upregulation of cytokines, which can be used as markers of inflammation. While most cytokines were not found to be influenced by aBL, there were a few that were significantly upregulated, including TIMP-1 in the female mice, and TIMP-1, MCSF, MCP-5, TNF RII, and CXCL1 [KC] in the males. The higher number of cytokines upregulated in the males than in females suggested that aBL induces a more diverse inflammatory response in the males. Interestingly, TIMP-1, which was found to be upregulated in both male and female mice, has been found to be associated with reduced inflammatory pain (Knight et al., 2019). This finding suggests the impact of aBL in reducing pain as a result of inflammation, which has been suggested in another study by Reuss et al. (2021). However, given the preliminary nature of the study, a deeper insight into the effect of aBL on inflammatory pain is warranted.

Immunohistochemical analysis revealed no indications of reduced cell proliferation or increased apoptosis in mouse skin following aBL exposure, suggesting aBL may not compromise wound healing. However, a previous study has reported that blue light does reduce the proliferation of human skin cells *in vitro* (Liebmann et al., 2010), through induction of differentiation of cells. It is possible that the *in vitro* paradigm may not necessarily reflect an *in vivo* situation, given that there exists a complex microenvironment within intact skin. In addition, in our study, we used aBL at 405 nm instead of 412–454 nm used by Liebmann et al. (2010), which may explain why we did not observe any impact of aBL on cellular proliferation. It is important to appreciate that our study is preliminary and requires further work to effectively understand the effect of aBL exposure on cellular proliferation at both *in vitro* and *in vivo* levels. Our findings also showed that aBL does not increase cell apoptosis *in vivo*, which is highly consistent with our previous works that used the TUNEL assay as an indicator of apoptosis. A recent clinically driven study by Cotter et al. (2021) also supports our findings, as they did not find aBL at 405 nm (55 J/cm^2^) to have any significant adverse effects on human skin. However, it is essential that further clinically driven studies using higher aBL exposures are carried out to identify a therapeutic window, prior to implementing aBL as a treatment option against *V. vulnificus* wound infections.

## Conclusion

Antimicrobial blue light is a highly effective approach against *V. vulnificus* infections. Additionally, aBL does not appear to have any adverse effects on mouse skin tissue or local inflammation, although further clinical studies are required to validate the safety of aBL in human patients.

## Data Availability Statement

The raw data supporting the conclusions of this article will be made available by the authors, without undue reservation.

## Ethics Statement

The animal study was reviewed and approved by Institutional Animal Care and Use Committee (IACUC) of the Massachusetts General Hospital.

## Author Contributions

CA, LL, XL, and TD conceived and designed the study. CA and LL carried out the experiments and performed data analysis. CA, LL, and TD wrote the manuscript. HM and RA edited the manuscript. All authors contributed to the article and approved the submitted version.

## Funding

This work was funded by the U.S. Department of Defense through the Military Medical Photonics Program (FA9550-20-1-0063) and the National Institute of Health (R01AI123312 to TD). CA was supported by the 2021 ASLMS Student Research Grant. LL was supported by the 2021 Bullock Postdoctoral Fellowship Program.

## Conflict of Interest

The authors declare that the research was conducted in the absence of any commercial or financial relationships that could be construed as a potential conflict of interest.

## Publisher's Note

All claims expressed in this article are solely those of the authors and do not necessarily represent those of their affiliated organizations, or those of the publisher, the editors and the reviewers. Any product that may be evaluated in this article, or claim that may be made by its manufacturer, is not guaranteed or endorsed by the publisher.
