# Supplementary material for: Antimicrobial Blue Light for Prevention and Treatment of Highly Invasive Vibrio vulnificus Burn Infection in Mice
Source: Front Microbiol. 2022 Jul 12;13:932466. doi: 10.3389/fmicb.2022.932466 (PMC9315199; doi:10.3389/fmicb.2022.932466)
Supplement: Supplementary file 1 [file Data_Sheet_1.pdf]

Supplementary Material

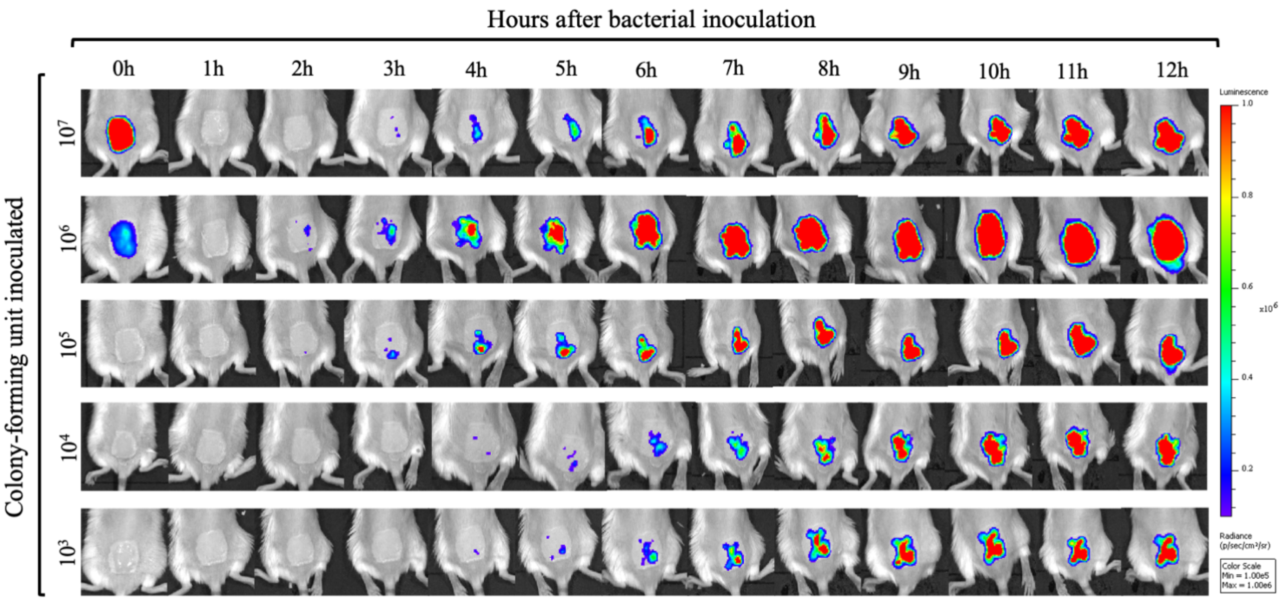

Supplementary Figure 1. Development of a mouse model of *Vibrio vulnificus* burn infection using a genetically engineered bioluminescent strain of *Vibrio vulnificus* (BCRC 81152; genotype pilA luxCDABE). Bioluminescence imaging was performed using an IVIS Lumina II *in vivo* Imaging System (PerkinElmer, US). Five (5) different inocula of *V. vulnificus* were applied to 5 mice, respectively. Bioluminescence images were taken at 1 h intervals for 12 h.

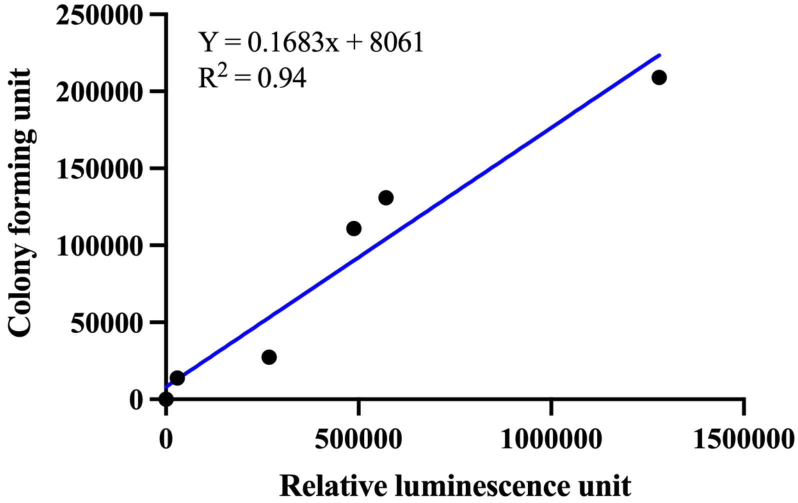

Supplementary Figure 2. Linear correlation between bacterial colony-forming units (CFU) and relative luminescence units (RLU) in mouse burns infected with a genetically engineered bioluminescent strain of *V. vulnificus* (BCRC 81152; genotype pilA luxCDABE) and treated with varying exposures of antimicrobial blue light.

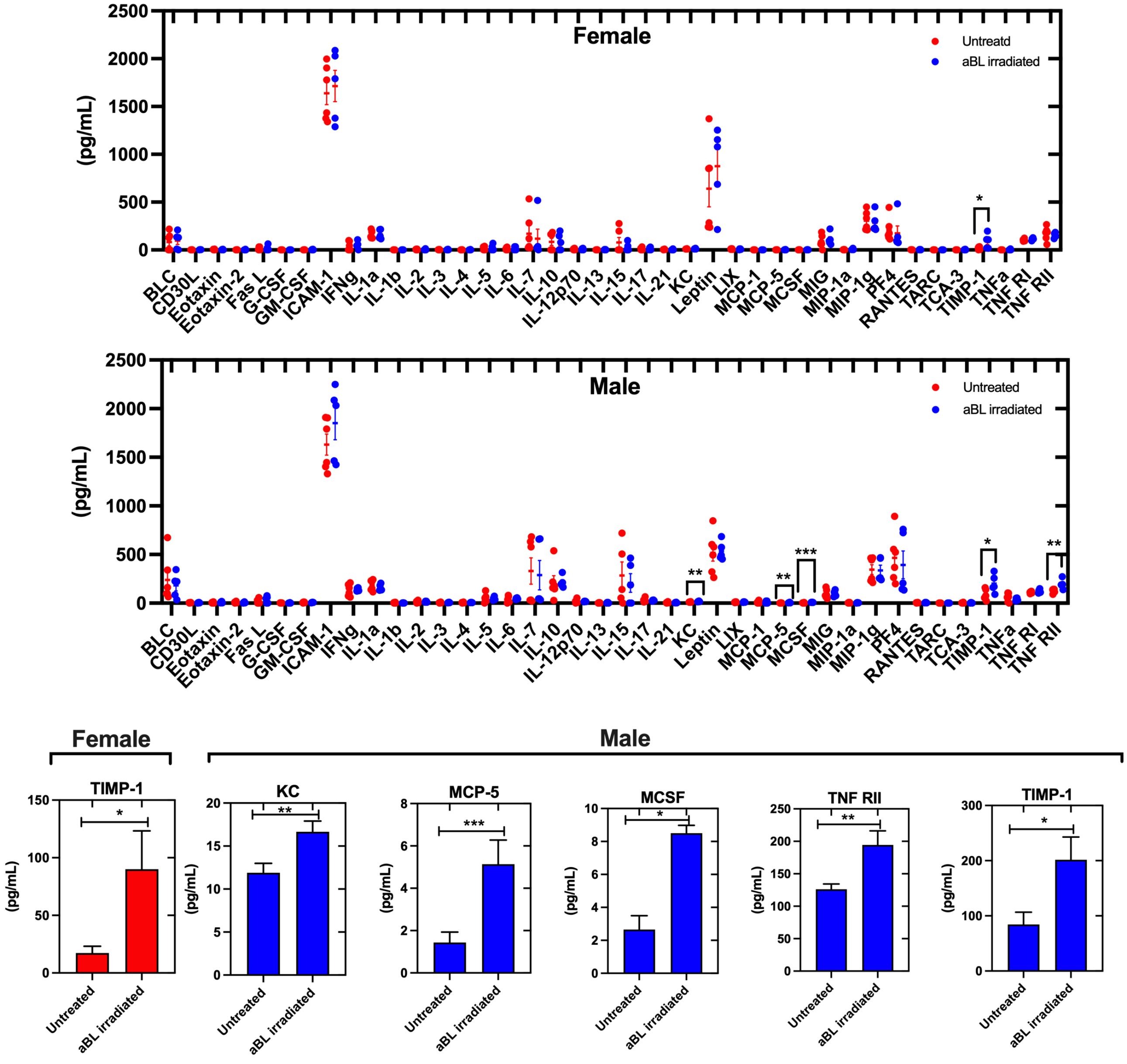

Supplementary Figure 3. Cytokine expression in response to no treatment or antimicrobial blue light irradiation (360 J/cm^2), which was analyzed from naïve mouse skin tissue isolated 24 hours after exposure. Cytokine concentrations were compared between groups using t-test. *P<0.05, **P<0.01, ***P<0.001.

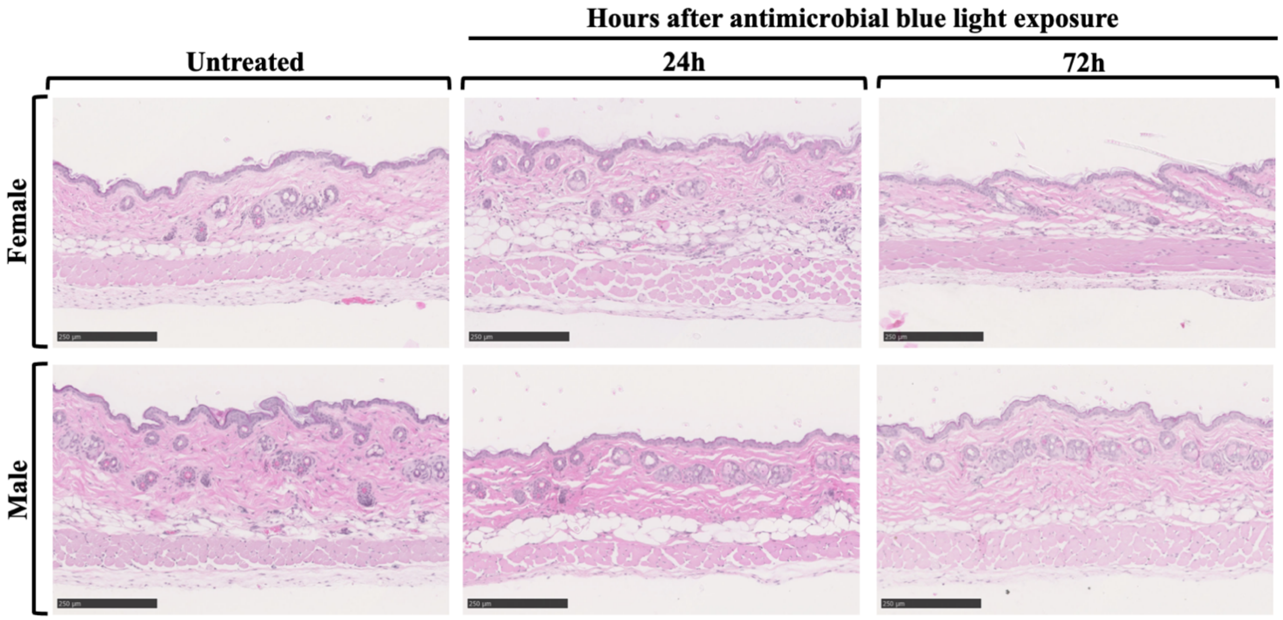

Supplementary Figure 4. Hematoxylin and eosin-stained histological sections of mouse naïve skin exposed to antimicrobial blue light (360 J/cm^2). Skin samples were collected after 24 and 72h of light exposure. Bar: 250 µm.

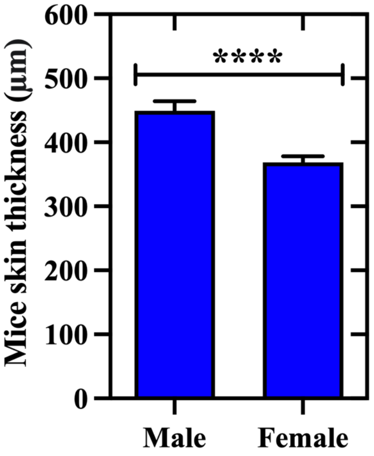

Supplementary Figure 5. Mouse skin thickness measurement was completed using the histological H&E samples. Analyses was performed in naïve mice skin (n=9) in 5 different regions of interest. Statistical significance between male and female skin was assessed unpaired T-test. ****$P<0.0001$.
